# Editorial: Celebration of DNA Day

**DOI:** 10.1186/s41065-024-00319-x

**Published:** 2024-05-06

**Authors:** Ramin Massoumi, Yongyong Shi

**Affiliations:** 1 Department of Laboratory Medicine, Translational Cancer Research, Faculty of Medicine, Lund University, 22381 Lund, Sweden; 2 Bio-X Institutes, Key Laboratory for the Genetics of Developmental and Neuropsychiatric Disorders (Ministry of Education), Shanghai Jiao Tong University, Shanghai, 20030 China; 3 Institute of Neuroscience, State Key Laboratory of Neuroscience, Center for Excellence in Brain Science and Intelligence Technology (CEBSIT), Chinese Academy of Sciences, Shanghai, 20031 China

As April unfolds, we are approaching DNA Day on April 25th, which marks one of the most pivotal moments in scientific history. This day serves not only as a celebration of a groundbreaking discovery, but also as a highlight of the intricate blueprint that shapes life as we know it - Deoxyribonucleic Acid (DNA).

**Figure:**
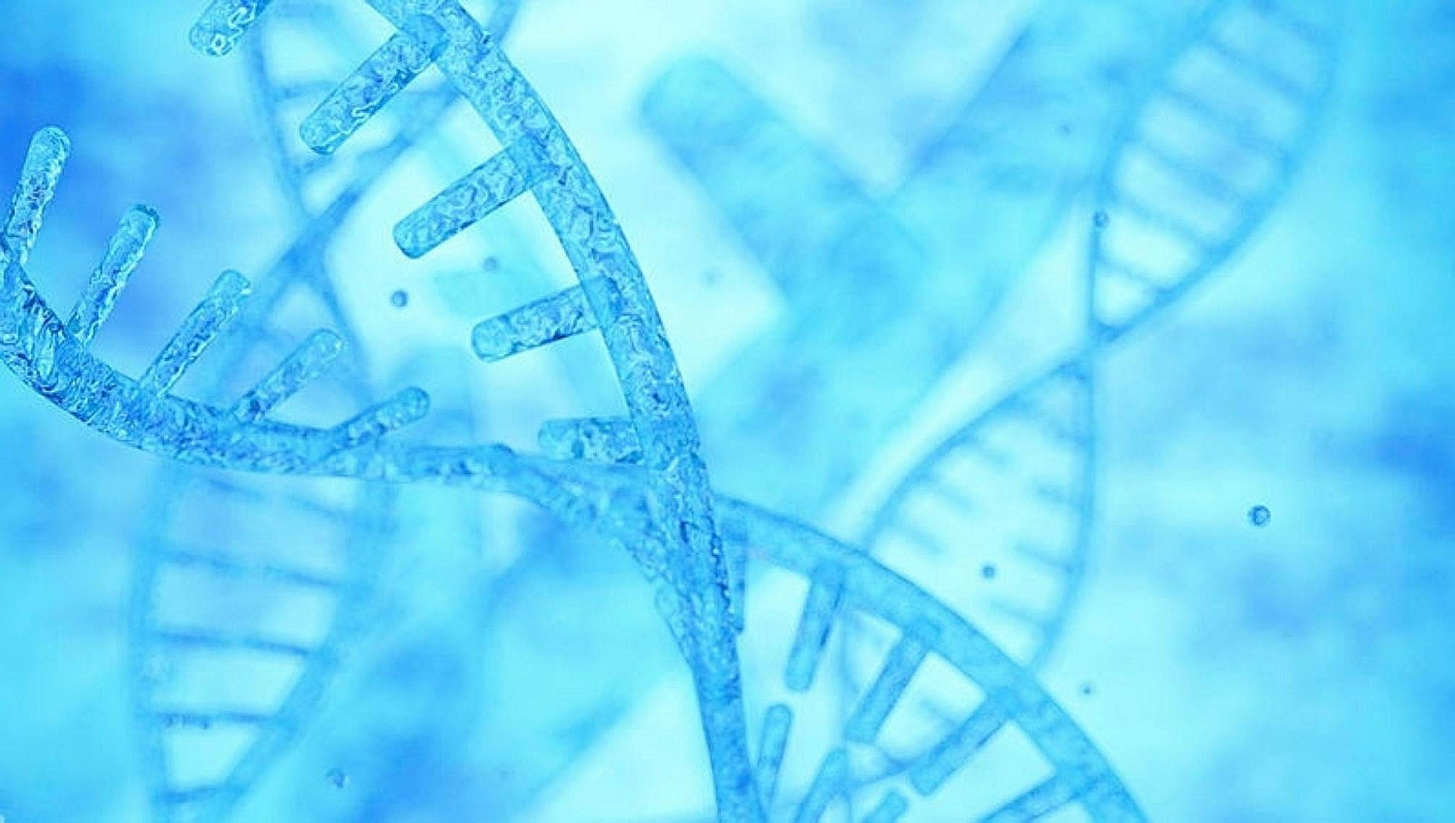
© asbe / Getty Images / iStock

In 1953, James Watson, Francis Crick, Rosalind Franklin, and Maurice Wilkins unveiled the double helix structure of DNA [1–3], which transformed our understanding of genetics and laid the foundation for numerous advancements in medicine, agriculture, forensics, and beyond. The discovery of DNA was akin to deciphering the code of life itself, offering a glimpse into the mysteries of inheritance, evolution, the very essence of our being, and the causes of genetic disorders.

Today, DNA Day is more than just a historical milestone. It is a celebration of the relentless pursuit of knowledge, the spirit of discovery, and the collaborative efforts of scientists from around the globe. It serves as a testament to what humanity can achieve when we come together to unravel the complexities of the natural world. Genetics continues to evolve at an unprecedented pace, fueled by technological advancements such as CRISPR gene editing, next-generation sequencing, high-throughput DNA synthesis, and precision medicine. These innovations promise to revolutionise healthcare, agriculture, conservation, and many other fields, offering solutions to some of the most pressing challenges facing humanity.

Notably, the *Hereditas* journal began publication in 1920, about 33 years before the discovery of DNA structures, by the Mendelian Society of Lund in Sweden [4–7]. In its long history, *Hereditas* has published significant papers in the field of genetics, such as the first discovery of the correct human chromosome count by Joe Hin Tijo and Albert Levan in 1956 [8]. Along with the technical surges based on the ever-improving understanding of DNA, the scope of *Hereditas* has evolved to publish groundbreaking research that expands our knowledge of genetics, genomics, and their myriad applications. From revealing complex inheritance patterns to exploring the role of genetics in evolution and disease, the contributions of authors, reviewers, and editors to our journal have been nothing short of remarkable.

However, with great power comes great responsibility. As we unlock the mysteries embedded in our DNA, it’s crucial to proceed cautiously, prioritizing ethical principles, safeguarding privacy, and promoting fair access to genetic insights, technologies, and advantages across diverse global communities. *Hereditas* remains committed to fostering discussions on these critical issues, providing a platform for diverse voices to shape the future of genetics research and its applications.

DNA Day serves as a poignant reminder of the complexity and endless potential of our genetic code. It’s a day to celebrate past achievements, present advancements, and future possibilities in the ever-evolving world of genetics. As we mark this special day, *Hereditas* extends its gratitude to the global community of researchers, readers, and supporters who continue to push the boundaries of what is possible in the genetics and genomics field.
